# A meta-analysis of the relationship between the RPE and well-being in adolescent athletes: the critical moderating role of well-being dimensions

**DOI:** 10.3389/fpsyg.2025.1698568

**Published:** 2025-11-12

**Authors:** Yimeng Gu, Yunpeng Liu, Long Cheng

**Affiliations:** 1 Wushu College, Henan University, Kaifeng, China; 2 Henan University, Kaifeng, China

**Keywords:** adolescent athletes, Rating of Perceived Exertion (RPE), well-being, meta-analysis, athlete monitoring

## Abstract

**Introduction:**

The relationship between the Rating of Perceived Exertion (RPE) and well-being in adolescent athletes is controversial, complicating effective athlete monitoring. This meta-analysis aimed to clarify this relationship and investigate the critical moderating role of well-being dimensions.

**Methods:**

Following the PRISMA guidelines, we systematically searched the Web of Science and PubMed, including 24 studies (57 effect sizes).

**Results:**

An initial analysis of the overall relationship revealed extremely high heterogeneity (I^2^= 85.6%), indicating that pooling all well-being dimensions is inappropriate. Subgroup analysis was decisive, identifying the nature of the well-being indicator as the key moderator. The RPE was strongly positively correlated with consumptive indicators (e.g., fatigue, delayed onset muscle soreness (DOMS); *r* = 0.51) but moderately negatively correlated with restorative indicators (e.g., sleep quality; *r* = −0.45), with the difference between these groups being highly significant (*p* < 0.0001). Sport type, age, and gender were not significant moderators. Although publication bias was detected (*p* = 0.014), sensitivity analyses confirmed the robustness of the crucial subgroup findings. The generalizability of these results may be limited as the included samples predominantly consisted of elite, male adolescent athletes.

**Conclusion:**

In conclusion, the RPE–well-being relationship is profound but context-dependent, driven by the nature of the well-being metric (consumptive vs. restorative). This provides a scientific basis for more precise athlete monitoring.

**Systematic review registration:**

CRD420251138178, https://www.crd.york.ac.uk/PROSPERO/view/CRD420251138178.

## 1 Introduction

Youth sport represents a complex environment where young athletes are typically exposed to high physical and psychological demands (Bergeron et al., 2015). As it is vital to avoid maladaptations to training, such as non-functional overreaching or injury, monitoring young athletes’ internal load and well-being status is critical (Standing et al., 2022). Perceptual scales are non-invasive and easy to use and have become very relevant in this regard (Rabbani et al., 2017). Many of these scales, such as the Rating of Perceived Exertion (RPE), used to quantify training load (Williams, 2017) and a wide range of well-being questionnaires (e.g., the Hooper scales) used to assess recovery status (Hooper and Mackinnon, 1995) are significantly influential (Foster et al., 2001). Perceptual scales are increasingly being viewed as useful tools for representing athletes’ psychophysiological state and can be conceptualized within a wider framework of emotional intelligence—that is, the ability of the athlete to recognize and manage their internal state in relation to sport-specific contexts (Lane et al., 2009).

While both the RPE and well-being are monitored concurrently in many settings, the precise relationship between them remains controversial and requires further clarification (Andrade et al., 2020). Well-being itself is a multi-faceted construct, commonly assessed through dimensions such as sleep quality, stress, fatigue, and delayed onset muscle soreness (DOMS) (Kellmann and Beckmann, 2018). It is currently unclear whether the correlation between the RPE and well-being is consistent across these different dimensions. For instance, does a high RPE score have the same relationship with a consumptive indicator such as fatigue as it does with a restorative indicator such as sleep quality? This lack of clarity makes it difficult for coaches and practitioners to interpret the combined data from these monitoring tools effectively.

The concurrent monitoring of the RPE and well-being is theoretically grounded in the classic stimulus–response model of athletic training. Within this framework, training load—captured effectively through the RPE—represents the external stimulus or stressor intended to provoke physiological adaptation (Halson, 2014). Well-being measures, in turn, provide an index of the athlete’s psychophysiological response to this stimulus, reflecting the ongoing balance between fatigue and recovery. The fundamental aim of athlete monitoring is to manage this delicate equilibrium, ensuring that training loads are sufficient to stimulate positive adaptation without exceeding the athlete’s adaptive capacity, which could otherwise result in non-functional overreaching, illness, or injury (Meeusen et al., 2013; Soligard et al., 2016). Therefore, a clear and predictable relationship between the perceived stimulus (RPE) and the resultant state (well-being) is not merely of theoretical interest but represents a practical necessity for effective training prescription and athlete welfare.

Therefore, the primary aim of the present meta-analysis was to systematically quantify the relationship between the RPE and subjective well-being in adolescent athletes. Specifically, our first objective was to determine the overall correlation between the RPE and well-being scores, as measured across four common dimensions: sleep quality, stress, fatigue, and DOMS. Our second, more crucial, objective was to investigate the sources of heterogeneity in this relationship by examining the moderating effects of several key “sport circumstances.” Based on the conceptual differences between the well-being indicators, we hypothesized that the nature of the well-being dimension itself (i.e., restorative vs. consumptive) would be the most significant moderator. We also exploratorily investigated whether other factors, such as sport type, gender, and age, also moderate the RPE–well-being relationship.

## 2 Research methods

The protocol for this meta-analysis was registered with the International Prospective Register of Systematic Reviews (PROSPERO), registration number CRD420251138178. The literature search was independently conducted by the first author and the corresponding author in strict accordance with the 《PRISMA 2020》 Statement. Relevant studies were retrieved through computer-based searches of the Web of Science and PubMed databases. The search strategy combined subject terms and free-text keywords, using Boolean operators (AND/OR). Key search terms included (“Adolescen*” OR “Youth”) AND (“Perceived Exertion” OR “RPE”) AND (“well-being” OR “sleep” OR “stress” OR “fatigue” OR “DOMS” OR “affect” OR “recovery”).

The inclusion criteria were as follows: (1) Study participants were adolescent athletes, defined as having a mean age between 12 and 18 years. (2) The study measured athletes’ Rating of Perceived Exertion (RPE) using a validated scale (e.g., Borg CR10 scale). (3) The study measured at least one subjective well-being indicator, specifically including sleep quality, fatigue, stress, or DOMS. (4) The study was an original observational study (e.g., cross-sectional or cohort study). (5) The study reported a correlation coefficient (Pearson’s r) between the RPE and a well-being indicator or provided sufficient data to calculate it.

The exclusion criteria were as follows: (1) Studies involving adult or child (pre-adolescent) athletes; (2) studies that did not measure the RPE or a relevant well-being indicator; (3) review articles, meta-analyses, case reports, conference abstracts, or non-peer-reviewed articles; and (4) studies that did not report a correlation coefficient or provide the necessary data for its calculation.

### 2.1 Data extraction

After the literature screening process, two researchers independently extracted data from the included studies using a standardized data extraction form. All extracted entries were cross-checked, and any disagreements were resolved through discussion or consultation with a third expert. The extracted information was categorized into two main types: (1) fundamental study characteristics, which included publication details (first author, publication year), participant characteristics (total sample size, mean age, percentage of male athletes), and study contexts (sport type, competition level), and (2) core data for effect size calculation, which included the correlation coefficient (*r*) between the RPE and each well-being indicator, the corresponding *p*-value, and the specific sample size (*N*) used for the correlation analysis. The full search strategies for all databases are provided in Appendix 1.

When studies reported non-parametric correlations (Spearman’s *ρ*) instead of Pearson’s *r*, the coefficients were retained and treated as equivalent, given that both indices converge in magnitude under large sample conditions and continuous data distributions (Bonett and Wright, 2000; Bishara and Hittner, 2015). Previous meta-analytic research in sport and exercise psychology has adopted this approach, as the difference between the two coefficients is typically negligible for behavioral data of this type. To ensure robustness, all correlation coefficients were Fisher’s z-transformed before analysis.

### 2.2 Methodological quality of the included studies

The methodological quality of the included literature was assessed using the JBI checklist, and the results indicated a high overall quality. As detailed in Appendix Table 1, all 24 included studies were rated as having a “low” risk of bias. Given the consistent high quality across all studies, no studies were excluded, and no sensitivity analyses based on study quality were deemed necessary.

### 2.3 Statistical analysis

All statistical analyses were conducted using the R software. The correlation coefficient (*r*) was used as the effect size and was Fisher’s z-transformed prior to pooling. A random effects model was primarily used to calculate the pooled effect sizes, given the potential for heterogeneity, which was assessed using the I^2^ statistic. Subgroup analyses and meta-regression were employed to investigate sources of heterogeneity. Finally, a systematic assessment of publication bias and the robustness of the findings was conducted using Egger’s regression test, the trim and fill method, and a leave-one-out analysis. The significance level for all statistical tests was set at a *p*-value of <0.05.

## 3 Results

### 3.1 Literature search and study characteristics

**Figure 1:**
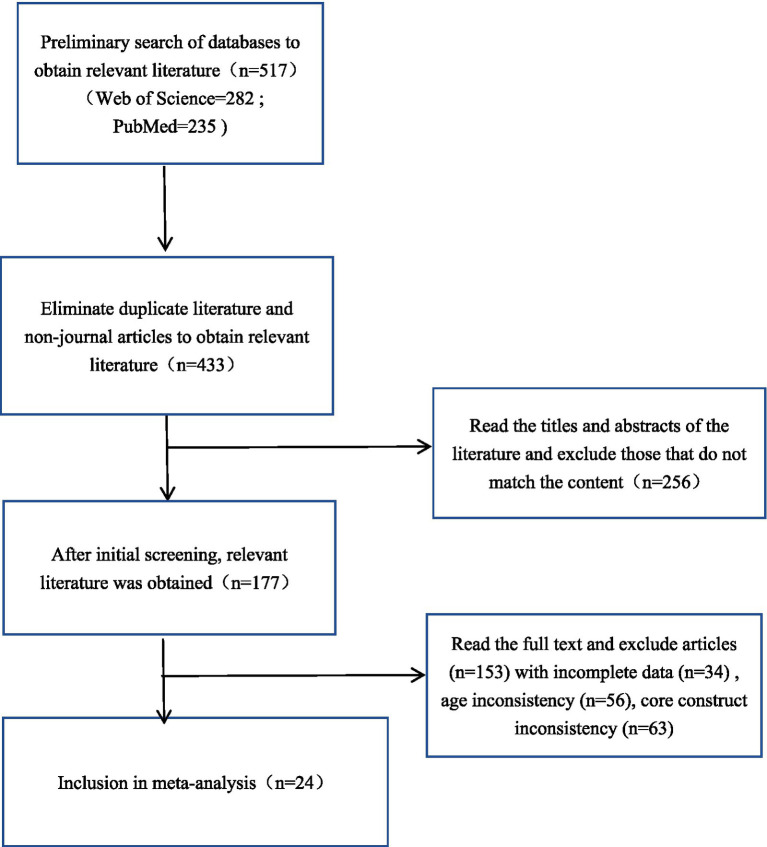
Literature screening flow chart.

The figure illustrates the literature screening process (Figure 1), resulting in the final selection of 24 studies for inclusion in the meta-analysis.

This meta-analysis ultimately included 24 studies that met the criteria from 427 studies, with a total of 57 independent effect sizes. The descriptive statistics of all included studies are shown in Table 1.

**Table 1:** Descriptive characteristics of the included studies (*N* = 57 effect sizes).

Characteristic	Category	Statistic
Publication year	Mean ± SD	2020.9 ± 3.0
	Range	2010–2025
Sample size	Mean ± SD	27.1 ± 17.7
	Range	12–98
Mean age	Mean ± SD	16.4 ± 0.7
	Range	14.1–17.6

Characteristic	Category	Quantity (*n*, %)
Well-being dimension	Sleep	15 (26.3%)
	Fatigue	13 (22.8%)
	Stress	11 (19.3%)
	DOMS/soreness	9 (15.8%)
	Other	9 (15.8%)
Sport type	Soccer	31 (54.4%)
	Combat sports	15 (26.3%)
	Other sports	11 (19.3%)
Gender composition	All men	40 (70.2%)
	Mixed gender	14 (24.6%)
	All women	3 (5.3%)
Competition level	Elite level	51 (89.5%)
	Non-elite level	6 (10.5%)

As shown in Table 1, the included studies were published between 2010 and 2025, with an average year of publication of September 2020 (SD = 3.0). The sample size ranged from 12 to 98 participants, with an average of 27.1 participants (SD = 17.7).

All participants were adolescent athletes (aged 12–18 years), predominantly male (70.2%), and primarily engaged in soccer, with the majority being elite athletes.

Regarding the core variables of greatest interest, sleep accounted for 26.3% (*n* = 15), fatigue 22.8% (*n* = 13), stress 19.3% (*n* = 11), and DOMS/soreness 15.8% (*n* = 9), representing a relatively even distribution.

### 3.2 Overall analysis of the RPE–well-being relationship and heterogeneity

**Figure 2:**
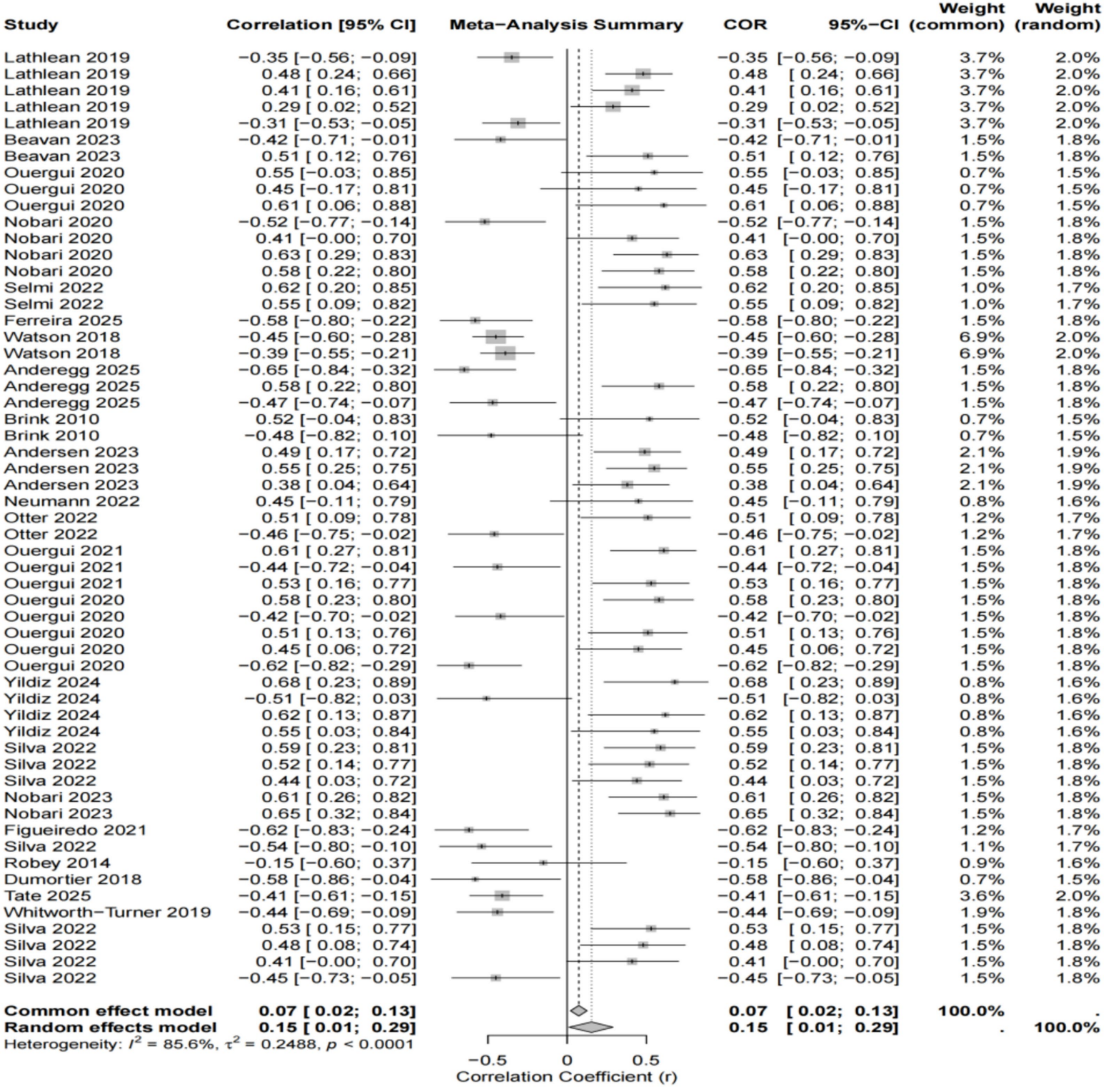
Forest plot of the overall relationship between the RPE and well-being (Lathlean et al., 2019; Beavan et al., 2023; Ouergui et al., 2020a,b; Nobari et al., 2020; Selmi et al., 2022; Ferreira et al., 2025; Watson and Brickson, 2018; Anderegg et al., 2025; Brink et al., 2010; Andersen et al., 2023; Neumann et al., 2021; Otter et al., 2022; Ouergui et al., 2021; Yildiz et al., 2024; Silva R. M. et al., 2022; Silva et al., 2022a,b; Nobari et al., 2023; Figueiredo et al., 2021; Robey et al., 2014; Dumortier et al., 2018; Tate et al., 2025; Whitworth-Turner et al., 2019).

Following the pooled analysis of all 57 effect sizes (see Figure 2), and given the high heterogeneity observed between the studies, the results of the random effects model were primarily considered. The findings indicated a weak but statistically significant positive correlation between the RPE and overall well-being (*r* = 0.15, 95% CI [0.01, 0.29], *p* = 0.033).

This result suggests that as the RPE increases, athletes tend to report worse negative well-being indicators (e.g., fatigue, soreness), although the strength of this overall association is very small.

Concurrently, the test for heterogeneity revealed that between-study heterogeneity was extremely high (I^2^ = 85.6%, Q-test *p* < 0.0001). This indicates that it is inappropriate to pool all dimensions of well-being together and that grouped statistics are required; therefore, conducting subgroup analyses is necessary.

### 3.3 Subgroup analyses and meta-regression

#### 3.3.1 Subgroup analysis by specific well-being dimension

To investigate the source of the high heterogeneity observed in the main forest plot, subgroup analyses were conducted for the specific dimensions of sleep, fatigue, DOMS/soreness, and stress.

As shown in Table 2, for the three dimensions reflecting physical load and consumption (fatigue, DOMS, and stress), the RPE demonstrated a significant positive correlation with each. The strongest correlation was observed for the fatigue dimension, followed by DOMS and stress. Notably, heterogeneity within each of these three subgroups was zero (I^2^ = 0.0%), indicating highly consistent results. For the sleep dimension, which reflects physical recovery, the RPE showed a significant moderate negative correlation, also with zero heterogeneity (I^2^ = 0.0%).

**Table 2:** Subgroup analysis of the relationship between the RPE and well-being, stratified by well-being dimension.

Subgroup	Number of observations	Correlation_r	95% confidence interval (CI)	*P*-value	Heterogeneity (I^2^)
Random effects model (sleep)	453	−0.45	[−0.52; −0.37]	<0.0001	I^2^ = 0
Random effects model (fatigue)	315	0.56	[0.48; 0.64]	<0.0001	I^2^ = 0
Random effects model (DOMS)	228	0.52	[0.41; 0.61]	<0.0001	I^2^ = 0
Random effects model (stress)	252	0.41	[0.30; 0.51]	<0.0001	I^2^ = 0

#### 3.3.2 Comparison of restorative versus consumptive well-being dimensions

**Figure 3:**
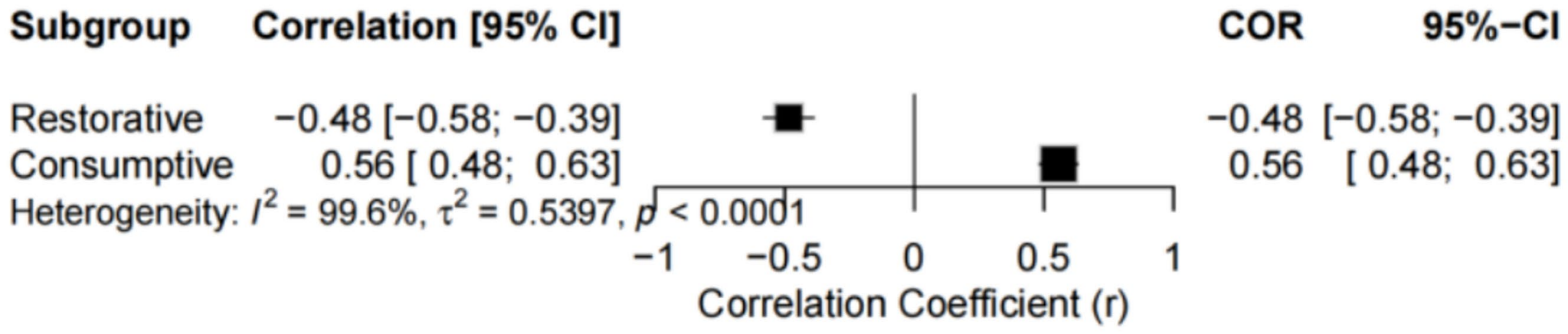
Forest plot of the final combined effect size of the recovery index and consumption index subgroups.

To further test the differences between these effects, a subsequent subgroup analysis was performed by classifying fatigue, DOMS, and stress into a “Consumptive” group and sleep into a “Restorative” group. The results of this comparison are presented in Figure 3 and Table 3.

**Table 3:** Subgroup analysis of the relationship between the RPE and well-being based on indicator properties.

Subgroup	Included studies	Correlation_r	95% confidence interval (CI)	Heterogeneity (I^2^)
Restorative	15	−0.45	[−0.52; −0.39]	0
Consumptive	33	0.51	[0.45; 0.56]	0
Test of differences between the groups	Q-value = 278.64, *p* < 0.0001			

The results of this subgroup analysis revealed that the relationship between the RPE and well-being is moderated by the nature of the well-being indicators themselves. Table 3 clearly presents the final comparative analysis results for these two major groups.

In the restorative indicator subgroup (*k* = 15), the RPE exhibited a significant moderate negative correlation (*r* = −0.45, 95% CI [−0.52, −0.37]). In stark contrast, in the consumptive indicator subgroup (k = 33), the RPE showed a strong, significant positive correlation (*r* = 0.51, 95% CI [0.45, 0.56]). Notably, heterogeneity within both of these conceptual groups was reduced to an ideal 0.0%, which indicates that our classification was appropriate.

Most critically, the test for subgroup differences provided decisive statistical evidence for this observation. The test results demonstrated that the difference between the effect sizes of the restorative and consumptive indicators was highly statistically significant (Q = 278.64, *p* < 0.0001).

This finding strongly confirms that the high heterogeneity observed in the initial overall analysis was primarily caused by the fundamental differences in the direction and magnitude of the effects across the various well-being dimensions. Therefore, the nature of the well-being indicator (restorative vs. consumptive) is the most central moderating variable in the relationship between the RPE and well-being.

#### 3.3.3 Exploratory analysis of other moderators (sport type, gender, and age)

After establishing the core moderating role of the well-being dimensions, we conducted further exploratory subgroup analyses to examine whether other study-level characteristics (such as sport type, gender composition, and age) affect the relationship between the RPE and well-being.

First, an analysis by sport type was conducted using the fatigue subgroup, which showed the strongest effect size, as an example to determine if the strong positive correlation between the RPE and fatigue varied according to sport type.

As shown in Table 4, a significant moderate-to-strong positive correlation between the RPE and fatigue was observed across all analyzed sport types. This indicates that this phenomenon is universal and not limited to a specific type of sport. Within the same dimension, the difference in *r*-values between soccer and combat sports was not substantial, which suggests that sport type itself is not a moderating variable.

**Table 4:** Subgroup analysis of the relationship between the RPE and fatigue stratified by sport type.

Subgroup	Number of observations	Correlation_r	95% confidence interval (CI)	*P*-value	Heterogeneity (I^2^)
Australian football	54	0.48	[0.24; 0.66]	0.0002	–
Soccer	187	0.57	[0.46; 0.67]	<0.0001	I^2^ = 0
Combat sports	74	0.61	[0.42; 0.74]	<0.0001	I^2^ = 0

Next, a subgroup analysis was performed on the sleep dimension, as shown in Table 5.

**Table 5:** Subgroup analysis of the relationship between the RPE and sleep by sport type.

Subgroup	Number of observations	Correlation_r	95% confidence interval (CI)	*P*-value	Heterogeneity (I^2^)
Soccer	198	−0.48	[−0.59; −0.36]	<0.0001	I^2^ = 0
Other sports	255	−0.43	[−0.53; −0.32]	<0.0001	I^2^ = 0

As shown in Table 5, the effect sizes for the “Soccer” subgroup and the “Other Sports” subgroup were very close, which once again verifies the preceding conclusion: the relationship between the RPE and well-being (for both consumptive and restorative indicators) demonstrated good consistency across different sport types. Based on the available data, there is no evidence suggesting that sport type is a key moderating factor in this relationship.

To further examine the moderating effects of continuous variables, we conducted separate meta-regression analyses using the proportion of male athletes and the mean age of athletes as predictors (Table 6).

**Table 6:** Meta-regression analysis of the relationship between the RPE and fatigue.

Moderator	Regression coefficient (*β*)	Standard error (SE)	95% confidence interval (CI)	QM test	*P*-value
Proportion of male athletes (%)	0.0004	0.0028	[−0.005; 0.006]	0.02	0.90
Average age (years)	−0.308	0.1009	[−0.229; 0.167]	0.09	0.76

The findings demonstrated that the percentage of male athletes did not significantly predict the strength of the association between the RPE and fatigue and that mean age did not significantly moderate the relationship either.

In conclusion, our exploratory analyses of the three potential moderators of sport type, gender composition, and age showed that this relationship between the RPE and well-being is robust across contexts, as we found no evidence of key moderators of this relationship.

### 3.4 Publication bias assessment

**Figure 4:**
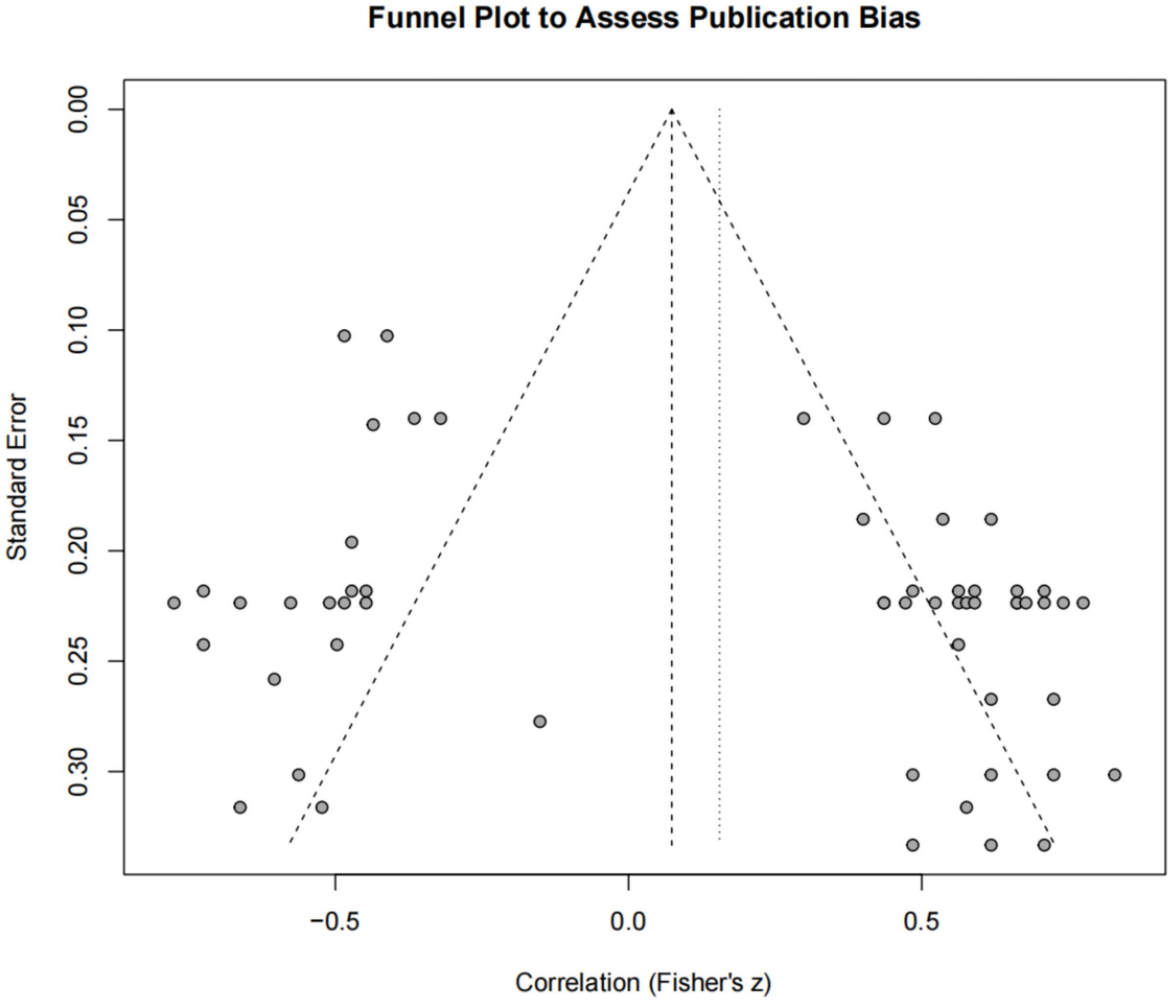
Funnel plot.

To assess the risk of publication bias in this study, a funnel plot was created using all 57 effect sizes, and Egger’s regression test was performed (Figure 4).

After visual inspection of the funnel plot, potential publication bias was suggested, and the results of Egger’s regression test provided some statistical support for this observation. The test results indicated that the funnel plot asymmetry was statistically significant (t = 2.55, *p* = 0.014). This indicates that studies reporting small or non-significant effect sizes may not have been adequately included in this meta-analysis, thereby the strength of the studies’ conclusions may have been somewhat inflated.

### 3.5 Sensitivity analyses

To address the issue of non-independent effect sizes originating from the same study, an additional sensitivity analysis was conducted using a robust variance estimation (RVE) model. When dependency among effect sizes was accounted for, the overall average correlation between the RPE and well-being was no longer statistically significant (*r* = 0.03, 95% CI [−0.16, 0.21], *p* = 0.78). This result differs from the initial pooled analysis (*r* = 0.15) and suggests that the weak positive association observed in the overall model may be an artifact of effect size dependence. This provides strong methodological support for our conclusion that indiscriminately pooling all well-being dimensions is inappropriate.

To examine the influence of individual studies on the subgroup results, we conducted leave-one-out sensitivity analyses separately for the “consumptive indicators” and “restorative indicators.” The results showed that sequential removal of any single study did not substantially alter the pooled effect size estimate. In the “consumptive” group, the pooled correlation coefficient remained between 0.55 and 0.58, while in the “restorative” group, it remained between −0.47 and −0.50. These results indicate that no single study disproportionately influenced the subgroup estimates.

Given that Egger’s test indicated potential publication bias, the trim and fill method was applied to further assess its impact. In the “consumptive” group, 12 potentially missing studies were imputed, resulting in an adjusted pooled correlation of *r* = 0.46 (from *r* = 0.51), which remained highly significant (*p* < 0.0001). In the “restorative” group, four studies were imputed, slightly adjusting the pooled correlation from *r* = −0.45 to *r* = −0.42, which also remained highly significant (*p* < 0.0001). Although the magnitude of the pooled effects decreased slightly, the direction and statistical significance were unchanged.

Taken together, these sensitivity analyses support the robustness of the subgroup findings, demonstrating that the observed negative correlation between the RPE and restorative indicators, as well as the positive correlation with consumptive indicators, remained resilient to potential data dependency and publication bias.

## 4 Discussion

As stated in the introduction, the relationship between the RPE and well-being in youth athletes has been described as complex and controversial. The present meta-analysis sought to clarify this relationship by systematically quantifying the effect and exploring key moderators. Our primary finding reveals that the controversial nature of this relationship is not random but is systematically driven by the intrinsic properties of well-being indicators themselves—specifically, whether they are consumptive or restorative in nature.

### 4.1 Interpretation of the main research findings

The most significant contribution of this meta-analysis is the clarification of the “controversial” relationship between the RPE and well-being reported in the literature. An initial overall analysis revealed a weak but significant positive correlation (*r* = 0.15), which was accompanied by extremely high heterogeneity (I^2^ = 85.6%). While this result should be interpreted cautiously on its own, it strongly demonstrates that indiscriminately pooling all well-being indicators is inappropriate.

Our core findings originate from the subgroup analysis, which perfectly explained the source of the high heterogeneity observed. The results clearly showed a bidirectional relationship between the RPE and well-being: for indicators reflecting physical consumption (i.e., fatigue, muscle soreness, and stress), the RPE exhibited a strong positive correlation (*r* = 0.51); whereas for the indicator reflecting physical recovery (i.e., sleep quality), the RPE showed a moderate negative correlation (*r* = −0.45). The difference between these two effects was highly statistically significant (*p* < 0.0001). The physiological and psychological implications of this finding are consistent: a higher level of perceived exertion always corresponds to a poorer state of well-being, whether this is manifested as an increase in consumption or a deficit in recovery. This suggests that in monitoring practice, well-being should not be viewed monolithically but must be precisely interpreted by distinguishing between the different dimensions of consumption and recovery. This sharp reduction in heterogeneity to I^2^ = 0% is not a statistical artifact but rather a logical consequence of the data structure. The extremely high heterogeneity observed in the initial model was inflated by pooling two fundamentally opposed relationships: the positive correlation between the RPE and the consumptive indicators and its negative correlation with the restorative indicators. Once these effects were analyzed separately, the main source of between-study variance was removed, resulting in near-zero residual heterogeneity. This outcome supports the validity and explanatory value of distinguishing between consumptive and restorative dimensions of well-being.

An important methodological consideration for this study was the risk of non-independent effect sizes, as several studies contributed more than one effect size. To test the influence of this factor on the overall result, we conducted a sensitivity analysis using an RVE model. In contrast to the weak but significant positive correlation observed in the initial pooled analysis (*r* = 0.15), the RVE model revealed that the overall average effect approached zero and was not statistically significant (*r* = 0.03, *p* = 0.78) after accounting for data dependency. This finding provides further methodological support for our argument—that the true and meaningful relationship between the RPE and well-being can only be understood by distinguishing between “consumptive” and “restorative” indicators in a subgroup analysis. Simply correlating the RPE with a monolithic “well-being” concept is unreliable, and this amalgamated relationship becomes non-significant when more rigorous statistical models are applied.

Furthermore, our exploratory analyses indicate that this core relationship demonstrates good consistency across different sport types (e.g., soccer vs. others), gender compositions, and age groups. These factors are not key moderators of this relationship, which further highlights that the nature of the well-being indicator itself is the sole factor that can explain the substantial heterogeneity observed among the studies.

### 4.2 Strengths and limitations of the study

The research methodology used in this study allows for strong confidence in the findings. The PRISMA 2020 guidelines were carefully followed to present a clear and repeatable procedure for the systematic search, screening, and data extraction of the literature. The quality of literature was high throughout our analysis, as confirmed using the trusted JBI tool, which rated all 24 studies included as “low risk of bias.” By analyzing our core findings via various sensitivity analyses, we confirmed that our key findings were robust. Both the trim and fill method and the leave-one-out method confirmed that our findings were not influenced by any of the studies included, nor would they produce misleading conclusions based solely on the possibility of publication bias.

However, despite these strengths, some limitations must be acknowledged. Evidence of significant publication bias (Egger’s Test, *p* = 0.014) is present, which suggests that the strength of the RPE–well-being relationship in the literature should be interpreted with caution since it could overestimate the true effect of the relationship. However, our sensitivity analyses indicate that the overall conclusions remain stable. The included studies predominantly featured elite, adolescent male soccer players. Consequently, while our findings are robust within this demographic, they may not directly apply to female athletes, non-elite competitors, or athletes from other sports with different physiological demands. Therefore, in the future, more samples of the population need to be studied. Finally, our analysis does not capture other potential restorative indicators described in the introduction, for example, “sport enjoyment,” which identifies an existing gap in the field and a noteworthy direction for future investigation.

### 4.3 Practical significance and future research directions

The findings of this study have significant practical implications for coaches, sport scientists, and rehabilitation specialists working with adolescent athletes. When monitoring the RPE, it should not be paired with a general “well-being” score; instead, specific consumptive indicators (e.g., fatigue) and restorative indicators (e.g., sleep) should be assessed concurrently to obtain a more comprehensive and accurate judgment of an athlete’s state. In areas related to youth sports, where relative physical maturation and emotional regulation vary significantly among participants, coaches must exercise caution when interpreting any perceptual data. During this preliminary stage of development, small increases or decreases in the RPE or well-being score may not reflect true physiological issues due to fluctuations in psychological states and may simply reflect normal changes. As such, coaches who engage in activity with adolescents are encouraged to view perceived exertion and well-being scores as ongoing assessments of their exercise engagement—utilizing these discussions to facilitate early identification of athlete mental overload, athlete disengagement, or difficulties in recovery. Training loads should be defined not only by average team responses but also by patterns in individual athlete responses over time. Including regular daily well-being check-ins (i.e., 1–2 min daily before each practice) can further provide coaches with the opportunity to identify earlier negative trends and consider adjustments to training loads or recovery strategies (i.e., training intensity, rest, or relaxation). A sustained high RPE accompanied by a sharp decline in sleep quality during training may serve as a stronger warning sign of overtraining than the presence of fatigue alone.

For future research, we propose the following directions: First, more studies are needed on female adolescent athletes and non-elite populations to verify the generalizability of this study’s conclusions. Second, future empirical research should incorporate a more diverse range of well-being indicators, particularly positive affective indicators such as “sport enjoyment,” to construct a more complete RPE–well-being relationship model (Perazzetti et al., 2025). Third, longitudinal tracking designs could be employed to investigate the causal relationship and dynamic trends between the RPE and well-being variables.

## 5 Conclusion

This meta-analysis suggests that a significant and context-dependent relationship exists between the RPE and well-being in adolescent athletes. The core driver of this relationship is the nature of the well-being indicator itself (consumptive vs. restorative), rather than the demographic characteristics of the athletes or their sporting environment. This finding provides an important scientific basis for more precise monitoring and protection of the physical and mental health of adolescent athletes.

## Data Availability

The original contributions presented in the study are included in the article/Supplementary material, further inquiries can be directed to the corresponding author.
