# Supplementary material for: A meta-analysis of the relationship between the RPE and well-being in adolescent athletes: the critical moderating role of well-being dimensions
Source: Front Psychol. 2025 Nov 12;16:1698568. doi: 10.3389/fpsyg.2025.1698568 (PMC12659709; doi:10.3389/fpsyg.2025.1698568)
Supplement: Supplementary file 1 [file Table_1.docx]

Web of Science

( TS=(adolescent* OR teen* OR youth*) AND TS=(athlete* OR player*) ) AND ( TS=("Rating of Perceived Exertion" OR "Perceived Exertion" OR RPE) ) AND ( TS=("well-being" OR "well being" OR sleep OR stress OR fatigue OR DOMS OR "delayed onset muscle soreness" OR "muscle soreness" OR myalgia OR mood* OR affect* OR recovery) )

PuBmed：

( (Adolescent[Mesh] OR adolescent*[tiab] OR teen*[tiab] OR youth*[tiab]) AND (Athletes[Mesh] OR athlete*[tiab] OR player*[tiab]) ) AND ( "Rating of Perceived Exertion"[tiab] OR "Perceived Exertion"[tiab] OR RPE[tiab] ) AND ( "well-being"[tiab] OR "well being"[tiab] OR Sleep[Mesh:NoExp] OR sleep[tiab] OR "Stress, Psychological"[Mesh] OR stress[tiab] OR Fatigue[Mesh] OR fatigue[tiab] OR Myalgia[Mesh] OR DOMS[tiab] OR "delayed onset muscle soreness"[tiab] OR "muscle soreness"[tiab] OR Affect[Mesh] OR mood*[tiab] OR affect*[tiab] OR recovery[tiab] )
